# Radial outer retina reflectivity (RORR) sign in *LAMP2* ‐associated retinopathy

**DOI:** 10.1111/aos.70178

**Published:** 2026-06-28

**Authors:** Rachael C. Heath Jeffery, Bohdan Kousal, Ulrike Grünert, Christine A. Curcio, Svetlana Cherepanoff, Tina M. Lamey, Michel Michaelides, Chrysanthi Tsika, Isabelle Chow, Omar A. Mahroo, Seungmin Lee, Martin Meliska, Milos Kubanek, Jakub Sikora, Siying Lin, Raymond C. B. Wong, Petra Liskova, Fred K. Chen

**Affiliations:** ^1^ Centre for Ophthalmology and Visual Science, the University of Western Australia Perth Western Australia Australia; ^2^ Ocular Tissue Engineering Laboratory, Lions Eye Institute Nedlands Western Australia Australia; ^3^ Royal Victorian Eye and Ear Hospital East Melbourne Victoria Australia; ^4^ Department of Ophthalmology, First Faculty of Medicine Charles University and General University Hospital in Prague Prague Czech Republic; ^5^ First Faculty of Medicine, Department of Pediatrics and Inherited Metabolic Disorders, Research Unit for Rare Diseases Charles University and General University Hospital in Prague Prague Czech Republic; ^6^ The University of Sydney, Faculty of Medicine, Save Sight Institute Sydney New South Wales Australia; ^7^ Department of Ophthalmology and Visual Sciences Heersink School of Medicine, University of Alabama at Birmingham Birmingham Alabama USA; ^8^ Sydpath, St Vincent's Hospital Darlinghurst New South Wales Australia; ^9^ Institute of Health Research, UNSW Darlinghurst New South Wales Australia; ^10^ St Vincent's Clinical School, UNSW Darlinghurst New South Wales Australia; ^11^ Australian Inherited Retinal Disease Registry and DNA Bank, Department of Medical Technology and Physics Sir Charles Gairdner Hospital Nedlands Western Australia Australia; ^12^ Moorfields Eye Hospital NHS Foundation Trust London UK; ^13^ UCL Institute of Ophthalmology, University College London London UK; ^14^ Section of Ophthalmology and Department of Twin Research and Genetic Epidemiology King's College London, St Thomas' Hospital Campus London UK; ^15^ Department of Physiology, Development and Neuroscience University of Cambridge Cambridge UK; ^16^ Pusan National University Hospital Busan South Korea; ^17^ Department of Cardiology Center for Inherited Cardiovascular Diseases, IKEM Prague Czech Republic; ^18^ First Faculty of Medicine Institute of Pathology, Charles University and General University Hospital Prague Czech Republic; ^19^ Faculty of Biology, Division of Evolution, Infection and Genomics School of Biological Sciences, Medicine and Health, University of Manchester Manchester UK; ^20^ Manchester Centre for Genomic Medicine, Saint Mary's Hospital, Manchester University NHS Foundation Trust Manchester Academic Health Science Centre Manchester Manchester UK; ^21^ Centre for eye Research Australia, Royal Victorian Eye and Ear Hospital Melbourne Victoria Australia; ^22^ Department of Surgery University of Melbourne Melbourne Victoria Australia

**Keywords:** Danon disease, dilated cardiomyopathy, external limiting membrane, hypertrophic cardiomyopathy, inherited retinal disease, LAMP2, Müller glia cells, optical coherence tomography, retina, X‐linked carrier

## Abstract

**Purpose:**

To describe the radial outer retina reflectivity (RORR) sign in patients carrying pathogenic variants in the X‐linked lysosome‐associated membrane protein‐2 (*LAMP2*) gene and to review the histologic characteristics of LAMP2 expression in the human retina.

**Methods:**

International multicentre observational case series and experimental laboratory study of patients with *LAMP2* deficiency. Baseline demographics and multimodal retinal imaging including colour and fundus autofluorescence (FAF), optical coherence tomography (OCT) with en face reconstruction and fluorescein angiography were obtained. Sections through the retina of a human donor eye were processed for immunofluorescence combining antibodies against *LAMP2* with markers for Müller glia, rod and cone photoreceptors. Sections were imaged using high‐resolution multichannel confocal microscopy. Assessment of en face OCT reflectivity profiles at 5–50 μm internal to the plane of external limiting membrane (ELM) was performed as well as histologic localisation of LAMP2.

**Results:**

We recruited 11 cases with pathogenic variants in *LAMP2* across five countries. Six cases demonstrated peripheral radial streaks of hyper‐ and hypopigmentation with UWF imaging. The RORR sign was best visualised 10–25 μm internal to ELM in 9 cases. We hypothesise the radial pattern to be secondary to X‐chromosome inactivation. LAMP2 immunoreactivity was observed internal and external to the ELM.

**Conclusion:**

Our findings expand the phenotypic spectrum of *LAMP2*‐associated retinopathy by demonstrating the RORR sign using en face OCT. Histologic sections revealed LAMP2 expression in the neuroretina.

**Clinical Relevance**

Danon disease (DD) belongs to a group of autophagy disorders with characteristic retinal findings. Following the recent phase 1 adeno‐associated virus gene therapy in DD, ophthalmologists and cardiologists must be aware of and recognise this retinal imaging sign so that patients are urgently directed to genetic services.

## 1 INTRODUCTION

Pathogenic variants in the lysosomal‐associated membrane protein‐2 (*LAMP2*) gene lead to Danon disease (DD) (Online Mendelian Inheritance in Man [OMIM] #300257) an X‐linked multisystem disorder (Nishino et al., 2000). DD classically presents with the triad of hypertrophic cardiomyopathy, skeletal myopathy and cognitive impairment (Nishino et al., 2000). Without an implanted defibrillator or heart transplant, affected individuals frequently die from sudden cardiac death before the age of 30 (Sugie et al., 2002). Whilst the differential diagnosis for a young patient presenting with a hypertrophic cardiomyopathy is wide, the retinal findings of *LAMP2* are useful (Dao et al., 2024; Hasegawa et al., 2022; Kousal et al., 2021). Hence a greater understanding and recognition of these ophthalmic signs can be lifesaving.

Schorderet et al. reported the presence of a pigmentary retinopathy in 2 hemizygous males and 1 female carrier (Schorderet et al., 2007). Meinert et al. also reported a pigmentary retinopathy in a heterozygous female harbouring a mosaic *LAMP2* variant (Meinert et al., 2019). However, the term ‘pigmentary retinopathy’ has been used to describe congenital rubella, single large‐scale mitochondrial DNA deletion syndromes, drug toxicities, post‐inflammatory diseases, and female carriers of X‐linked disorders such as ocular albinism and choroideraemia (CHM) (Emmanuele et al., 2022; Faure et al., 2022; Khan et al., 2018). Fundus autofluorescence (FAF) changes have also been described in LAMP2 deficiency (Kousal et al., 2021; Narayan et al., 2024; Thompson et al., 2016). More recently, greater loss of the near‐infrared FAF signal as compared to short‐wavelength FAF has been reported (O'Neil et al., 2022; Schmitz‐Valckenberg et al., 2011). In addition to a pigmentary retinopathy, hyperreflective lesions in the outer nuclear layer (ONL) on optical coherence tomography (OCT) have been described in 10 *LAMP2* cases of which 7 were female (Kousal et al., 2021). However, due to tissue patterns of X‐chromosome inactivation females with *LAMP2* deficiency have shown a more variable presentation compared to males. The hyperreflective lesions observed on OCT remain inconsistent with previous histological work in which LAMP2 was only seen in the RPE with no expression in the neuroretina (Schorderet et al., 2007).

Herein, we describe the Radial Outer Retina Reflectivity (RORR) sign in *LAMP2‐*associated retinopathy in the largest case series to date. Importantly, we also re‐examine LAMP2 in the neurosensory retina. Our study better aligns the clinical presentation of LAMP2 with the underlying histology. We propose ophthalmologists may play a pivotal role in examining young patients with a cardiomyopathy to support an accurate molecular diagnosis.

## 2 MATERIALS AND METHODS

### 2.1 Study design

This was an international multicentre, observational case series of patients with *LAMP2*‐associated retinopathy and an experimental laboratory study. The study protocol adhered to the tenets of the declaration of Helsinki and received approval from local ethics committees of the participating institutions: University of Western Australia (2021/ET000151), Sir Charles Gairdner Osborne Park Health Care Group (RGS04985) and the institutional review board of the General University Hospital in Prague (34/19). UK approvals were from Moorfields Eye Hospital and the Northwest London Research Ethics Committee. All patients or their legal guardians provided signed, informed consent prior to inclusion in the study. A search was initially performed in the database of the Western Australian Retinal Degeneration (WARD) study and major referral sites across the globe were later contacted for participation.

### 2.2 Study participants

Eleven cases (1 male and 10 females) with a clinical and molecular diagnosis of *LAMP2‐*associated retinopathy were recruited across different international sites including Australia, South Korea, the United Kingdom and the Czech Republic. Cases were included based on the presence of a pathogenic variant in the *LAMP2* gene with or without the typical systemic features of DD. Genetic testing was performed where available with next generation sequencing or Sanger Sequencing of peripheral blood DNA to identify a likely disease‐causing variant in the *LAMP2* gene. Table 1 shows the demographic data and ocular findings in 11 cases with *LAMP2* variants. A control healthy subject, two choroideraemia carriers and one ocular albinism carrier were also recruited for comparison.

**TABLE 1:** Participant demographics and *LAMP2* variants.

ID	Country	Sex	Age (years)	VAR logMAR	VAL logMAR	Refraction R	Refraction L	OCT	OCTA Optovue	OCTA Spectralis	FAF	FFA	Coding variant^a^	Protein consequence^a^	Family history germline or mosaic
1	UK	Female	14	0.06	0.04	−6.00/−1.25 × 180	−5.00/−2.75 × 15	✔	✔	✔			c.427del	p.(Ile143Serfs*5)	Positive Germline
2	UK	Female	12	0.00	0.00	0	0	✔	✔	✔			c.1039del	p.(Thr347Profs*4)	Positive Germline
3	UK	Male	16	0.00	0.00	N/A	N/A	✔		✔	✔		^b^ arr[GRCh38] Xq24 (120 437 686_120 509 321) × 0.	p.?	Negative Somatic mosaic by NGS
4	Ukraine	Female	21	1.00	1.00	0/−0.75	+0.75/−1.25	✔		✔	✔		c.470_471insTA	p.(Thr158Lysfs*26)	Positive Germline
5	Korea	Female	26	0.22	0.22	−2.25/−5.75	−2.00/−5.75	✔			✔	✔	c.928G>A	p.[Val310Ile, Lys289Phefs*26]^c^	Positive Germline
6	Australia	Female	27	0.00	0.00	0	0	✔	✔	✔	✔	✔	c.928G>A	p.[Val310Ile, Lys289Phefs*26]^c^	Negative Somatic mosaic by NGS
7^d^	Czechia	Female	28	0.00	0.00	Plano/−0.75 × 11	+0.75/−1.25 × 143	✔		✔	✔		c.398‐312_*7360del	p.(Gly133Aspfs*32)	Positive Germline
8^d^	Czechia	Female	31	0.24	0.22	−4,00/−3.00	−4.25/−3.00	✔		✔			c.418del	p.(Leu140Phefs*8)	Positive Germline
9^d^	Czechia	Female	36	0.20	0.00	+1.50/+2.50	+0.25/+2.50	✔		✔	✔		c.718C>T	p.(Gln240*)	Positive Germline
10^d^	Czechia	Female	36	0.00	0.02	−0.25/−1.00	−0.25/−1.25	✔		✔			c.445_449del	p.(Asp149Phefs*2)	Positive Germline
11	Korea	Female	52	0.00	0.00	+1.25/−1.75	+1.25/−2.50	✔			✔	✔	c.928G>A	p.[Val310Ile, Lys289Phefs*26]^c^	Positive Germline

Abbreviations: FAF, fundus autofluorescence; FFA, fundus fluorescein angiography; logMAR, logarithm of minimum angle of resolution; Mosaic, refers to non‐uniform presence of the variant in the somatic sample tested by NGS; N/A, not available; NGS, next generation sequencing; OCT, optical coherence tomography; OCTA, optical coherence tomography angiography; VAL, visual acuity left; VAR, visual acuity right.

^a^ *LAMP2* coding reference sequence (RefSeq) is NM_002294.3. Described variants are considered causative for *LAMP2*‐associated retinopathy. >; substitution, del; deletion, ins; insertion, *; stop codon, fs; frameshift, (); predicted/not experimentally confirmed protein consequence.

^b^ SNP array analysis using microarray with ~1.1 million probes across the genome showing an interstitial deletion of 0.072 Mb from band 24 in the long arm of the NC_000023.11 (X chromosome), between base pair coordinates 120 437 686 and 120 509 321, encompassing the 5′ end of *LAMP2* (OMIM 309060) and its exons 1–8.

^c^ c.928G is the last nucleotide of exon 7. This substitution leads to exon 7 skipping which introduces a frameshift and a premature termination codon (Bertini E, Donati MA, Cassandrini D, et al. Phenotypic heterogeneity in two unrelated Danon patients associated with the same LAMP‐2 gene mutation. Neuropediatrics 2005;36(5):309–13).

^d^ These cases are published in Kousal, B., Majer, F., Vlaskova, H., Dvorakova, L., Piherova, L., Meliska, M., et al. (2021) Pigmentary retinopathy can indicate the presence of pathogenic LAMP2 variants even in somatic mosaic carriers with no additional signs of Danon disease. Acta Ophthalmologica, 99(1):61–68.

A human eye from a 36‐year‐old female donor with no known eye disease was obtained postmortem from the Lions New South Wales Eyebank at the Sydney Eye Hospital with consent and ethics approval from The University of Sydney Human Research Ethics Committee (HREC# 2012/2833). The eye was enucleated 0.5 h after death, and the posterior eyecup was fixed in 2% paraformaldehyde in 0.1 M phosphate buffer for 40 h.

### 2.3 Outcome measures

All 11 cases underwent comprehensive ophthalmic examination including best corrected visual acuity (BCVA) in logarithm of the minimum of angle resolution (logMAR), subjective refraction, standard slit‐lamp biomicroscopy including dilated retinal assessment. Depending on availability, pseudocolour (Optos PLC, Dunfermline, United Kingdom) or true colour (Clarus 700, Carl Zeiss Meditec AG, Jena, Germany), fundus autofluorescence (FAF), fundus fluorescein angiography (FFA), OCT and OCT angiography (OCTA) images were also obtained. The 10° × 10° (3 × 3 mm) OCTA (512 B‐scans, 6 μm interscan separation, 4 images averaged) was acquired with the Spectralis (Heidelberg Engineering GmbH, Heidelberg, Germany) while the 6 × 6 mm OCTA was acquired with the Optovue‐XR (Avanti, Optovue. Inc., Fremont, California, United States of America).

### 2.4 Image analysis

The 3 × 3 mm en face OCT reflectivity maps were created by extracting the 2D reflectivity profile of the Spectralis OCTA cube at the level of the external limiting membrane (ELM). The spatial distribution of hyperreflective lesions within the outer nuclear layer (ONL) was assessed by moving the en face plane at 5 μm intervals up from the ELM towards the junction between ONL and outer plexiform layer (OPL). This was approximately 50 μm internal to the ELM. These reflectivity profiles were then compared to a healthy control subject. Two independent graders (RCHJ & FKC) determined the levels at which the reflectivity profile showed the RORR sign (Data S1). The 6 × 6 mm en face OCT reflectivity maps were generated by moving the choriocapillaris slab to the distal portion of the ONL encompassing the ELM.

### 2.5 Statistical analysis

Collected quantitative data were presented in tables as mean and standard deviation (SD). In the case of qualitative variables, data were reported as percentages.

### 2.6 LAMP2 expression and staining in the human retina

Vibratome sections through the macula of the human donor eye underwent processing for immunofluorescence and high‐resolution light microscopy as described previously (Lee et al., 2019; Masri et al., 2021, 2025). The sections were processed using a mouse monoclonal antibody against LAMP2 (H4B4‐c developed by August, J.T./Hildreth, J.E.K.) which was obtained from the Developmental Studies Hybridoma Bank, created by the Neuro Informatics, Computational and Data Analysis study section of the National Institutes of Health, and maintained at the University of Iowa, Department of Biology, Iowa City. Sections were double labelled with antibodies against LAMP2 and antibodies made in rabbit against Müller glia (CRALBP, Abcam ab243664), calcium binding protein calbindin (CaBP‐D28k Swant, CB38, lot: 5.5), to label cones or against S‐antigen (gift from Leo Peichl) to label rod photoreceptors. Secondary antibodies (made in donkey) were coupled to the fluorophores Alexa 594 or Alexa 488 (Jackson Immunoresearch Laboratories, Westgrove, PA, USA). High‐resolution confocal microscopy and differential interference contrast (DIC) microscopy were performed. Using previously published transcriptome data (Lukowski et al., 2019), the expression levels of *LAMP2*, *RLBP1*, *CALB1* and *SAG* were plotted for each retinal cell type as previously described (De Silva et al., 2021; Jiang & Mahroo, 2021). Expression levels for the same cell types were depicted with the same colours.

## 3 RESULTS

### 3.1 Participant demographics

This study included 11 *LAMP2* cases (10 (91%) were female and 1 (9%) was male). The mean ± SD (median, IQR, range) age was 28 ± 11 (27, 24–34, 14–52) years (Table 1). The mean ± SD (median, IQR, range) BCVA (logMAR) was 0.17 (0, 0–0.22, 0–0.24) and 0.16 (0, 0–0.22, 0–0.22) in the right and left eye respectively. The mean spectacle refraction was low myopia with −1.50D and −1.50D spherical equivalent in the right eye and left eye, respectively (Table 1). Clinical features of cases 7, 8, 9 and 10 were previously published (Kousal et al., 2021) and the multimodal imaging of cases 5 and 11 had been reported (Yang et al., 2024). Ocular and systemic symptoms and signs for each case are shown in Table 2.

**TABLE 2:** Ocular and systemic symptoms and signs.

Case ID	Ocular symptoms	Systemic symptoms	Age of symptom onset	Cardiac signs	Muscle signs	Brain signs
1	None	None	N/A	Hypertrophic cardiomyopathy	No skeletal muscle weakness	Normal intellect
2	None	None	N/A	No history of cardiac disease	No skeletal muscle weakness	Normal intellect
3		Cough, fatigue, nausea, collapsed due to cardiac failure		Hypertrophic cardiomyopathy	No skeletal muscle weakness	Normal intellect
4	None	Weakness and collapse, preceded by headache and dizziness	21	Extensive fibrotic changes of the left ventricle, pacemaker implantation at 21 years.	No skeletal muscle weakness	Normal intellect
5		None	N/A	No history of cardiac disease	No skeletal muscle weakness	Normal intellect
6	None	None	N/A	No history of cardiac disease	No skeletal muscle weakness	Normal intellect
7	None	Breathlessness, NYHA functional class II, syncopal episode and non‐sustained ventricular tachycardia	11	Hypertrophic cardiomyopathy, preexcitation in ECG, dual‐chamber cardioverter‐defibrillator	No skeletal muscle weakness	Normal intellect
8	None	Breathlessness, NYHA functional class III, pain due to liver congestion	28	Dilated cardiomyopathy, heart transplantation at 24 years.	No skeletal muscle weakness	Normal intellect
9	None	Breathlessness, NYHA functional class III, decompensated heart failure	10	Hypertrophic cardiomyopathy, preexcitation in ECG, developed end‐stage heart failure during pregnancy, heart transplantation at 27 years.	No skeletal muscle weakness	Normal intellect
10	None	Breathlessness, NYHA functional class II, palpitations, non‐sustained ventricular tachycardias	28	Dilated cardiomyopathy, diagnosed during a family screening	No skeletal muscle weakness	Normal intellect
11	Photopsia and blurred vision			Dilated cardiomyopathy, heart transplantation at 48 years.	No skeletal muscle weakness	Normal intellect

### 3.2 Molecular analysis

A likely disease‐causing variant in *LAMP2* was identified in each case (Table 1). Nine of the 11 cases had a known family history of DD whilst two were isolated cases with de novo somatic mosaicism. Case 3, the only male, had a de novo large deletion of approximately 0.072 Mb; arr[GRCh38] Xq24[120 437 686_120 509 321] × 0, encompassing exons 1–8 (of 9) detected via single nucleotide polymorphism array using a microarray with 1.1 million probes across the genome in the blood. Case 6 was a female with no family history and a de novo variant, c.928G>A, (both parents were negative for this variant) reported to weaken the 5′‐splice acceptor site resulting in exon 7 skipping (Too et al., 2017). Using target enrichment (capture) and Next Generation Sequencing, a Molecular Vision Panel (v21.1) with an average coverage of ~500 reads and at least 30× coverage in > 96% of the panel found 9–10% of the reads contained this variant. Cases 5 and 11 were from the same family and carried the same splice variant. The remaining 8 cases had nonsense (*n* = 1, case 9), small deletions (*n* = 4), large deletions (*n* = 2, cases 3 & 7) or insertions (*n* = 1, case 4) variants. All were predicted to lead to nonsense mediated decay. For comparison of the hyper and hypo‐pigmentary peripheral streaks, we included one carrier of ocular albinism harbouring a splice variant in the *GPR143* gene (NM_000273.3), c.360G>A, p.(Ala120=), and two female carriers of choroideremia (aged 38 and 61 years), each harbouring a distinct loss‐of‐function variant in the *CHM* gene (NM_000390.4), c.1479 T>C, p.(Cys493*) and c.685dup, p.(Ile229Asnfs*2), respectively.

### 3.3 Multimodal imaging

UWF‐Optos pseudocolour and FAF imaging were available for six of the 11 cases (5 female and 1 male). These cases were compared to one choroideremia (*CHM*) carrier and one X‐linked ocular albinism (*GPR143*) carrier in Figure 1. Peripheral radial hypo‐ and hyper‐pigmented streaks were noted in all *LAMP2* cases. These changes were most pronounced in a 52‐year‐old female (case 11), the oldest case within our cohort and a 16‐year‐old male (case 3). In contrast to the hypo‐ and hyper‐pigmented streaks observed in the choroideremia (*CHM*) and ocular albinism (*GPR143*) carriers, there was additional speckled hyperautofluorescence (hyperAF) in *LAMP2*. In case 5, this speckled hyperAF was localised predominantly to the posterior pole as opposed to cases 2 and 6 in which the speckled hyperAF was observed outside the posterior pole. Figure 2 demonstrates UWF‐Optos pseudocolour imaging of the peripheral retina in six *LAMP2* cases, two *CHM* carriers and one *GPR143* carrier. Whilst *LAMP2* cases showed linear hypopigmented streaks, the *CHM* carriers showed a branching reticular pattern and the *GPR143* carrier showed more regular, densely packed hypopigmented streaks. Coexistent peripheral drusen were observed in case 6. Figure 3 depicts two cases carrying *LAMP2* pathogenic variants with pseudocolour, FAF and FFA comparisons. FFA highlighted well‐defined bands of radial and speckled hyperfluorescence consistent with RPE window defects. This area of hyperfluorescence appears more extensive in size as compared to the hypoAF changes in the middle panel. The lower panels of Figure 3 show the peripheral retina on infrared reflectance and OCT.

**FIGURE 1:**
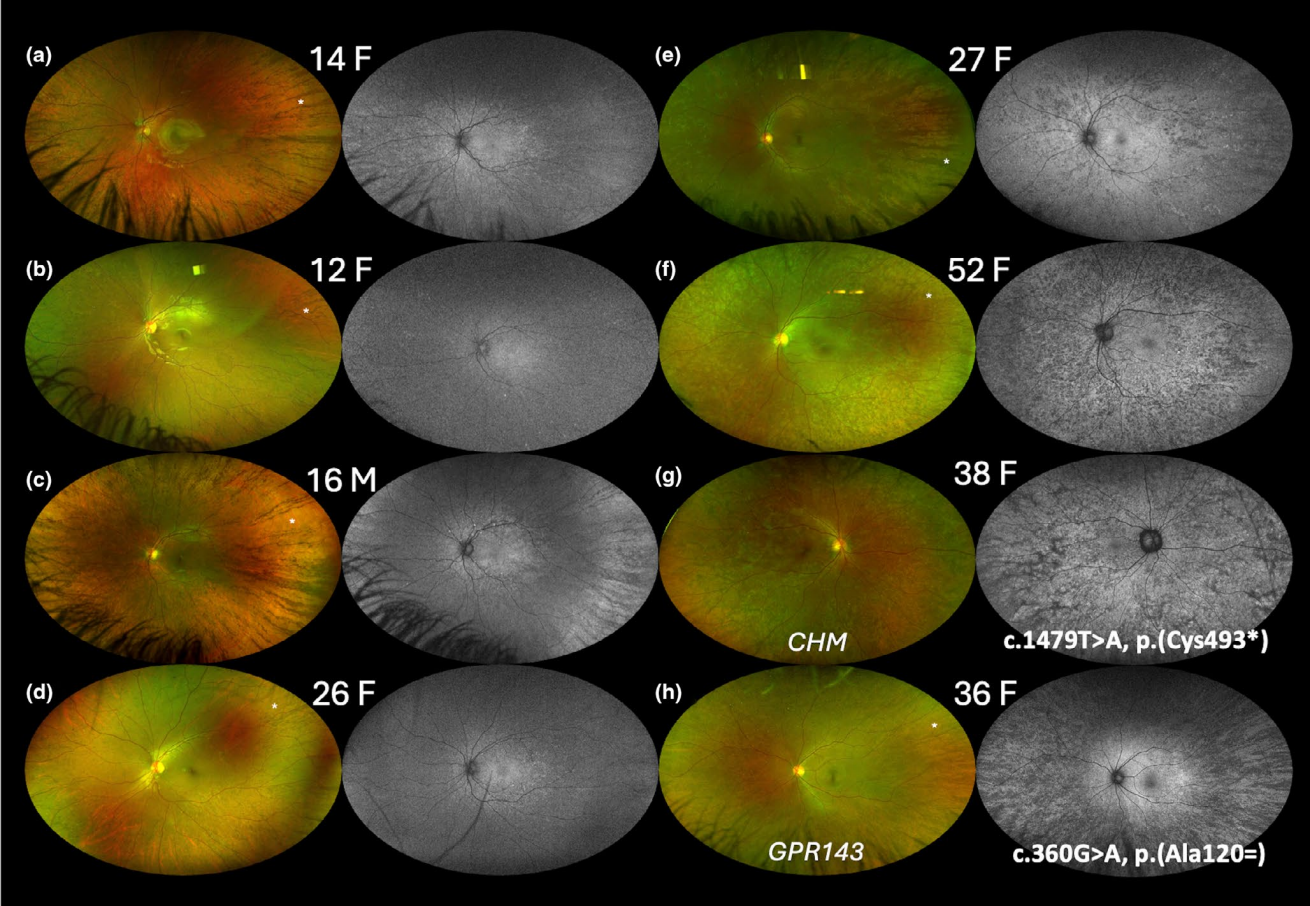
Ultrawide field pseudocolour and fundus autofluorescence Optos imaging for six of the 11 cases with pathogenic *LAMP2* variants from 12 to 52 years (a–f) and one choroideremia (*CHM*) carrier (g) and one ocular albinism (*GPR143*) carrier (h) for comparison. All *LAMP2* cases demonstrated peripheral radial hyperpigmented linear streaks which became hypopigmented with increasing age. In contrast the CHM carrier (c.1479 T>A) showed a branching reticular pattern of hyperpigmentation and the *GPR143* carrier (c.360G>A) exhibited a finer pattern of long, thin and densely packed hyper and hypopigmented streaks (white star).

**FIGURE 2:**
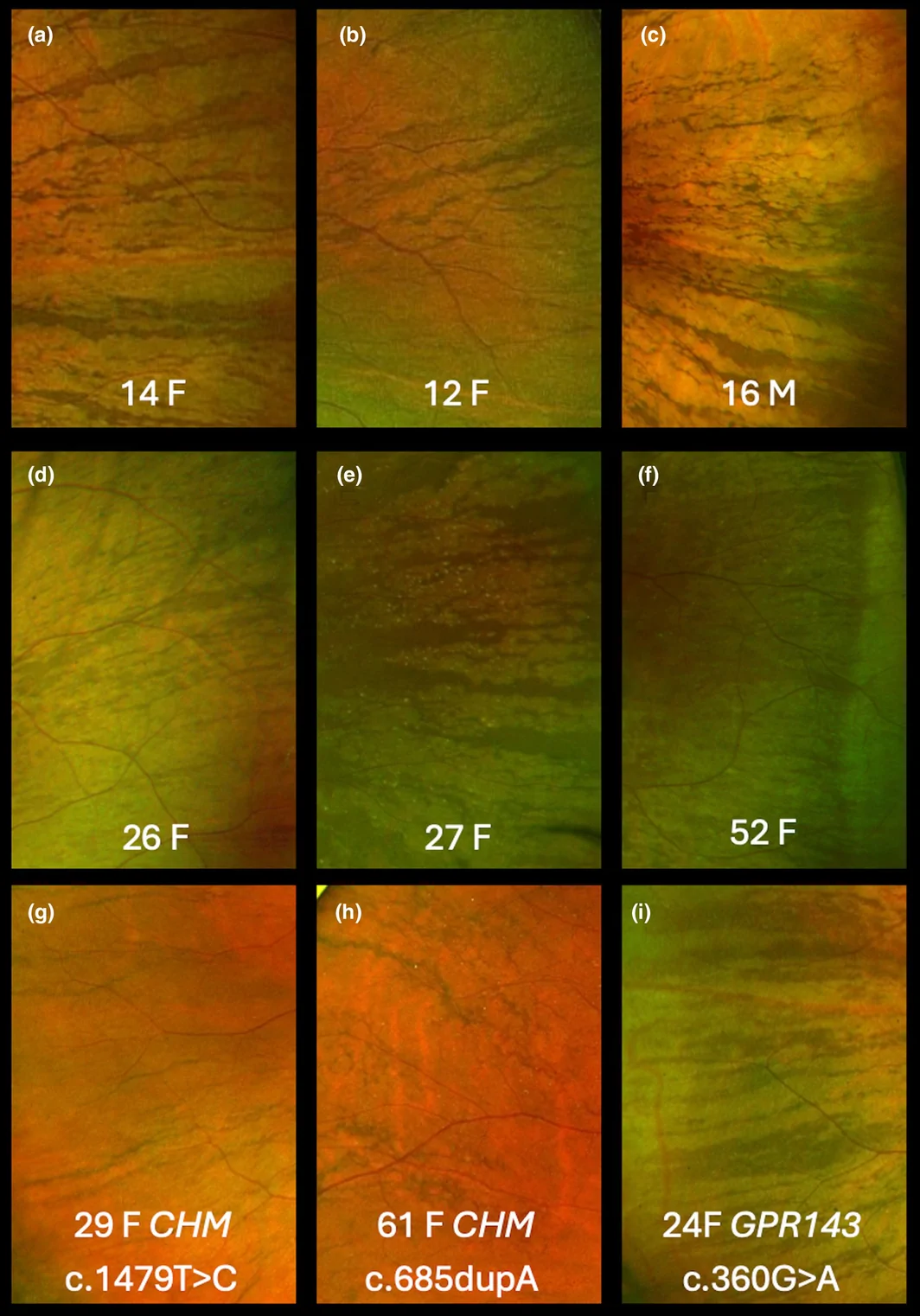
Higher magnification view of the peripheral retina using pseudocolour Optos images in six cases with pathogenic *LAMP2* variants (a–f). This contrasts with two *CHM* carriers (g: C.1479 T>C, h: C.586dupA) showing a reticular pattern of hyperpigmentation and one *GPR143* carrier (i) showing densely packed hyper and hypopigmented linear streaks.

**FIGURE 3:**
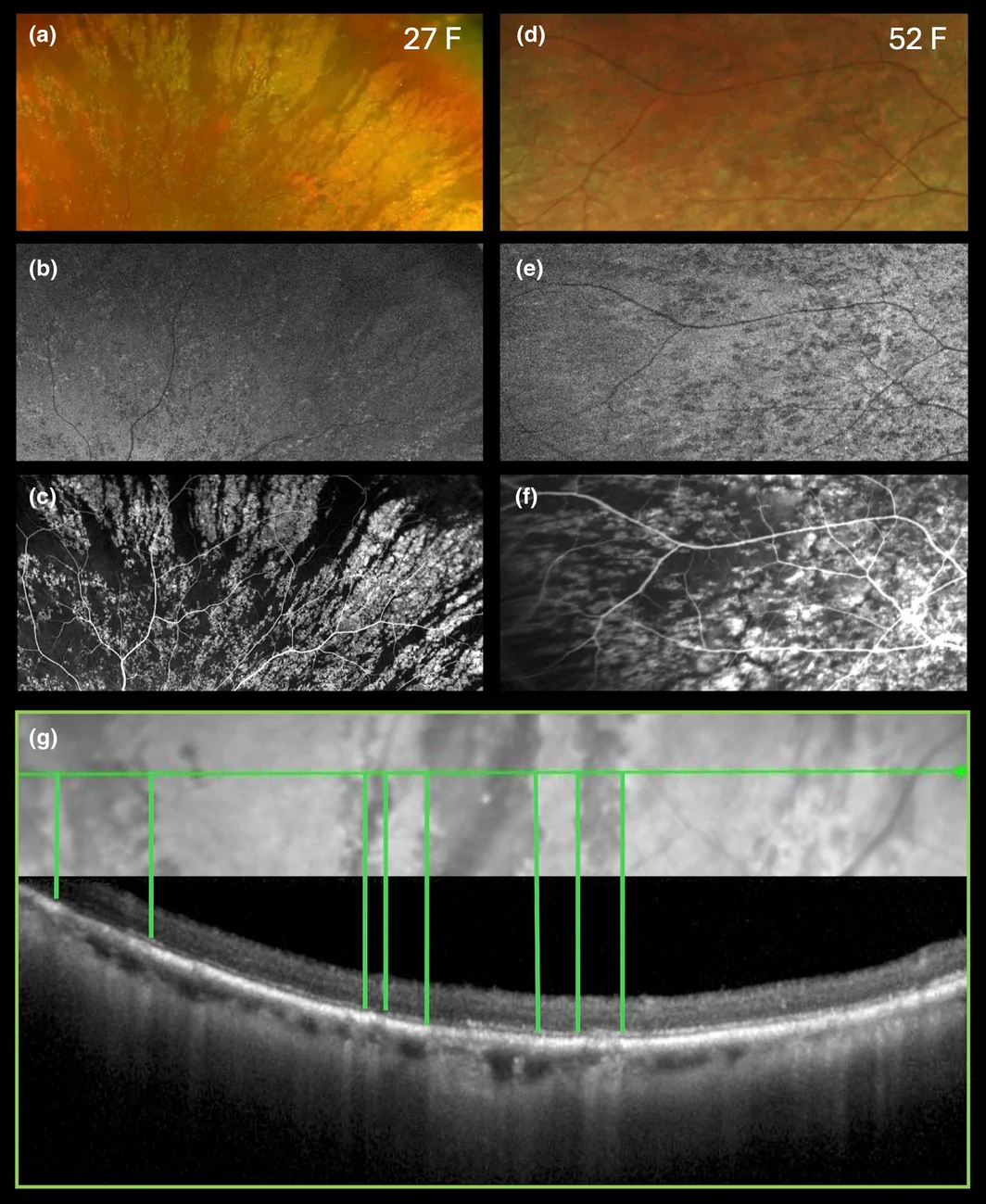
Ultrawide field Optos pseudocolour (a, d), fundus autofluorescence (b, e) and fluorescein angiography (c, f) of two cases with pathogenic *LAMP2* variants, a 27‐year‐old female (a–c) and a 52‐year‐old female (d–f) demonstrating peripheral linear streaks and grape‐like clusters of hyperfluorescence consistent with RPE window defect. Infrared reflectance imaging from the 27‐year‐old female showed the peripheral hypopigmented streak was aligned with regions of reduced reflectivity in the RPE and choroidal transmission on optical coherence tomography (g).

OCT en face reflectivity maps in the central 3 × 3 mm zone were available for nine of the 11 cases. Maps were generated 0 to 50 μm internal to the ELM at 5 μm intervals and showed central abnormal hyperreflectivity within the central 1 mm zone with a radial profile extending beyond 0.5 mm eccentricity (Data S1). B‐scans for all nine cases with en face are shown in Data S1. This radial reflectivity pattern within the ONL resembled the hypo‐ and hyperpigmentary streaks in the peripheral retina. We called this the Radial Outer Retina Reflectivity (RORR) sign. Table 3 shows the agreement between graders 1 and 2 for detecting the RORR sign. The RORR sign was visualised between 10 and 25 μm internal to the ELM in all cases by both graders. Figure 4 demonstrates the RORR sign at 20 μm internal to the ELM as compared to a healthy control (panel J). In Figure 5 en face reflectivity maps covering the central 6 × 6 mm zone and their corresponding OCT segmentation are shown for 3 cases compared to a control.

**TABLE 3:** Extent of radial outer retinal reflectivity (RORR) sign in consecutive enface images within the outer nuclear layer.

Case ID	Grader 1 (μm)^a^	Grader 2 (μm)^a^	Regions of overlap (μm)^a^	Slabs overlapping	Vertical span within the ONL (μm)^a^
1	10–35	10–35	10–35	6	25
2	15–35	10–35	15–35	6	25
3	5–35	5–40	5–35	7	30
4	10–30	10–35	10–30	5	20
6	5–25	5–30	5–25	5	20
7	5–40	5–40	5–40	8	35
8	5–30	10–30	10–30	5	20
9	5–30	10–35	10–30	5	20
10	5–30	10–40	10–30	5	20
Slab with sign common across the cohort	10–25	10–30	10–25	4	15

Abbreviation: ONL: outer nuclear layer.

^a^ Distance at which the slab is internal to the external limiting membrane.

**FIGURE 4:**
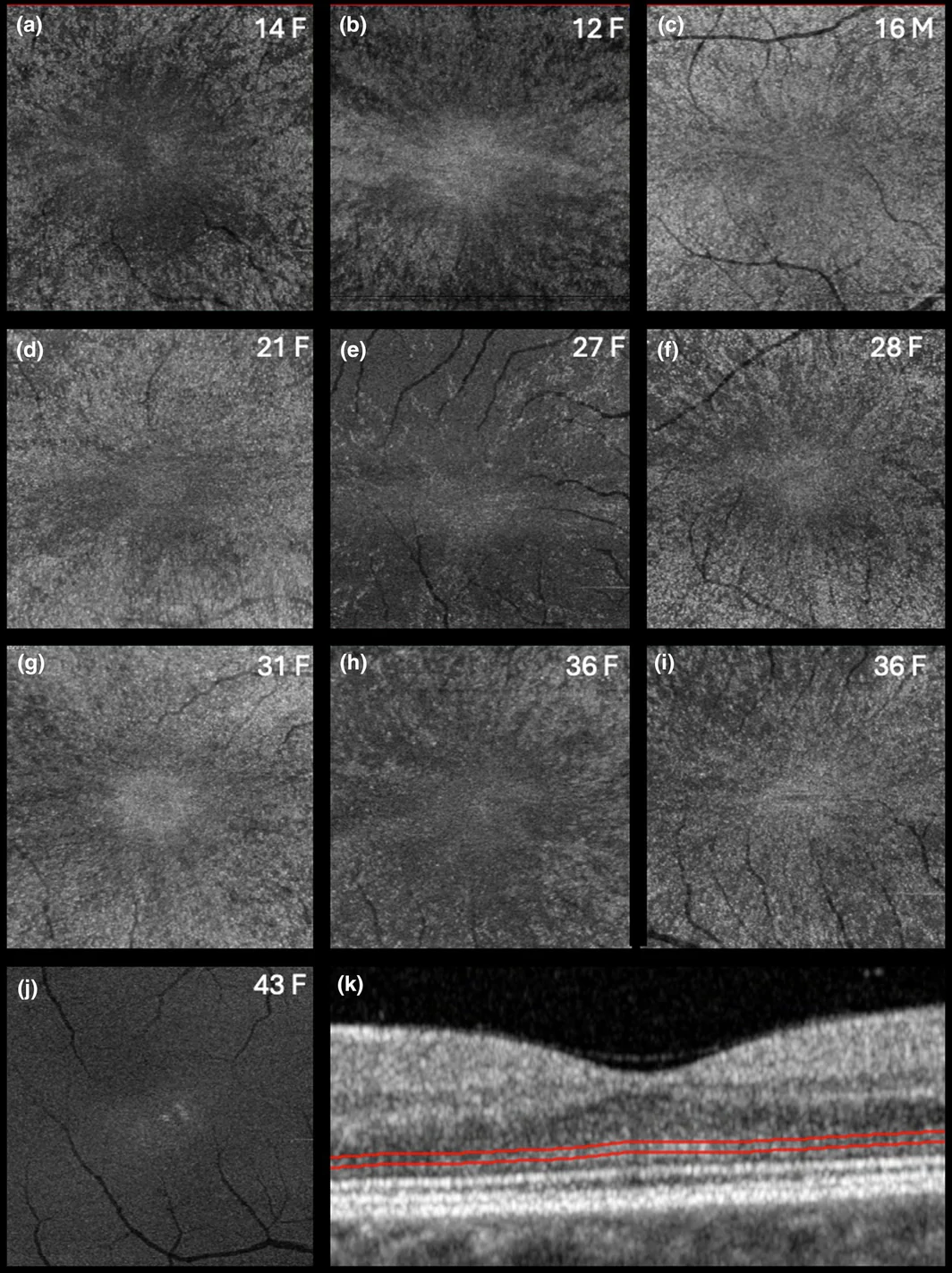
Spectralis 3 × 3 mm optical coherence tomography (OCT) en face reflectance maps at 20 μm internal to the external limiting membrane (ELM) for nine of the 11 cases with pathogenic *LAMP2* variants at increasing age (a–i) and one healthy control (j). OCT imaging with segmentation at the level of the ELM and 20 μm internal to the ELM (k). All *LAMP2* cases demonstrate the radial outer retina reflectivity (RORR) sign.

**FIGURE 5:**
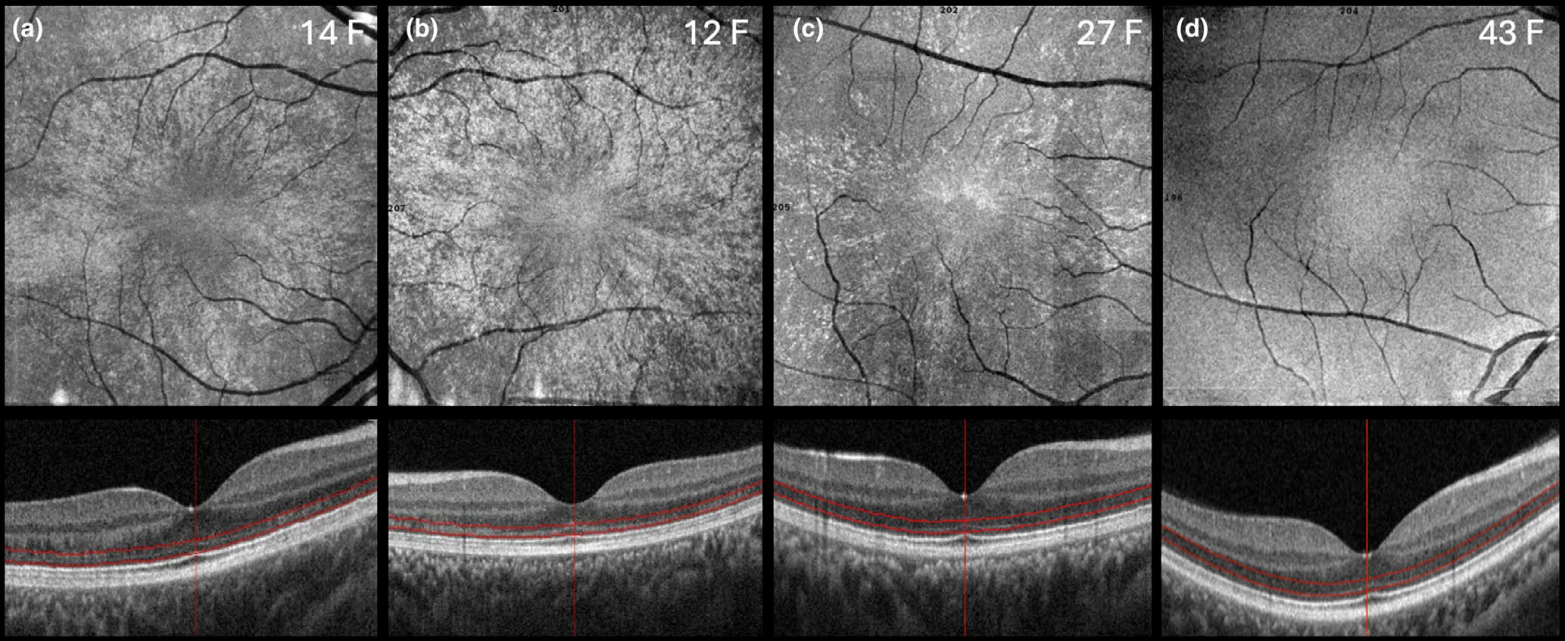
Optovue‐XR 6 × 6 mm en face reflectance maps generated from a slab segmented from the mid outer nuclear layer (ONL) to the external limiting membrane (ELM) showing the radial outer retinal reflectivity (RORR) sign extended beyond the central 3 × 3 mm region in three cases with pathogenic *LAMP2* variants (a–c). A control subject showed uniform reflectivity in the same slab (D).

### 3.4 LAMP2 staining profile

Immunohistochemistry analysis of human retinae showed expression of LAMP2 in the neuroretina (Figure 6). Müller glia (CRALBP), rod (SAG) and cone (Calbindin) photoreceptor markers were stained alongside LAMP2. DIC and standard microscopy images were obtained to demonstrate colocalisation and show tissue architecture including unstained elements (Figure 6). LAMP2 expression was observed in the RPE (not shown) and the Müller glia apical domain which might represented the apical cytoplasm of the Müller cell glia, although no clear co‐localisation with CRALB was observed. LAMP2 expression did not co‐localise with the cone marker Calbindin (CaBP) nor with the rod marker S‐antigen. This was consistent with single‐cell transcriptome data showing that LAMP2 expression was significantly higher in Müller glia than in cones, rods, bipolar or amacrine cells (Lukowski et al., 2019). Based on single cell transcriptome data available at the University of Iowa, the highest level of LAMP2 expression was found in Müller glia and RPE with some also found in vascular endothelial cells and pericytes (Figure 7) (Voigt et al., 2020; Yan et al., 2020).

**FIGURE 6:**
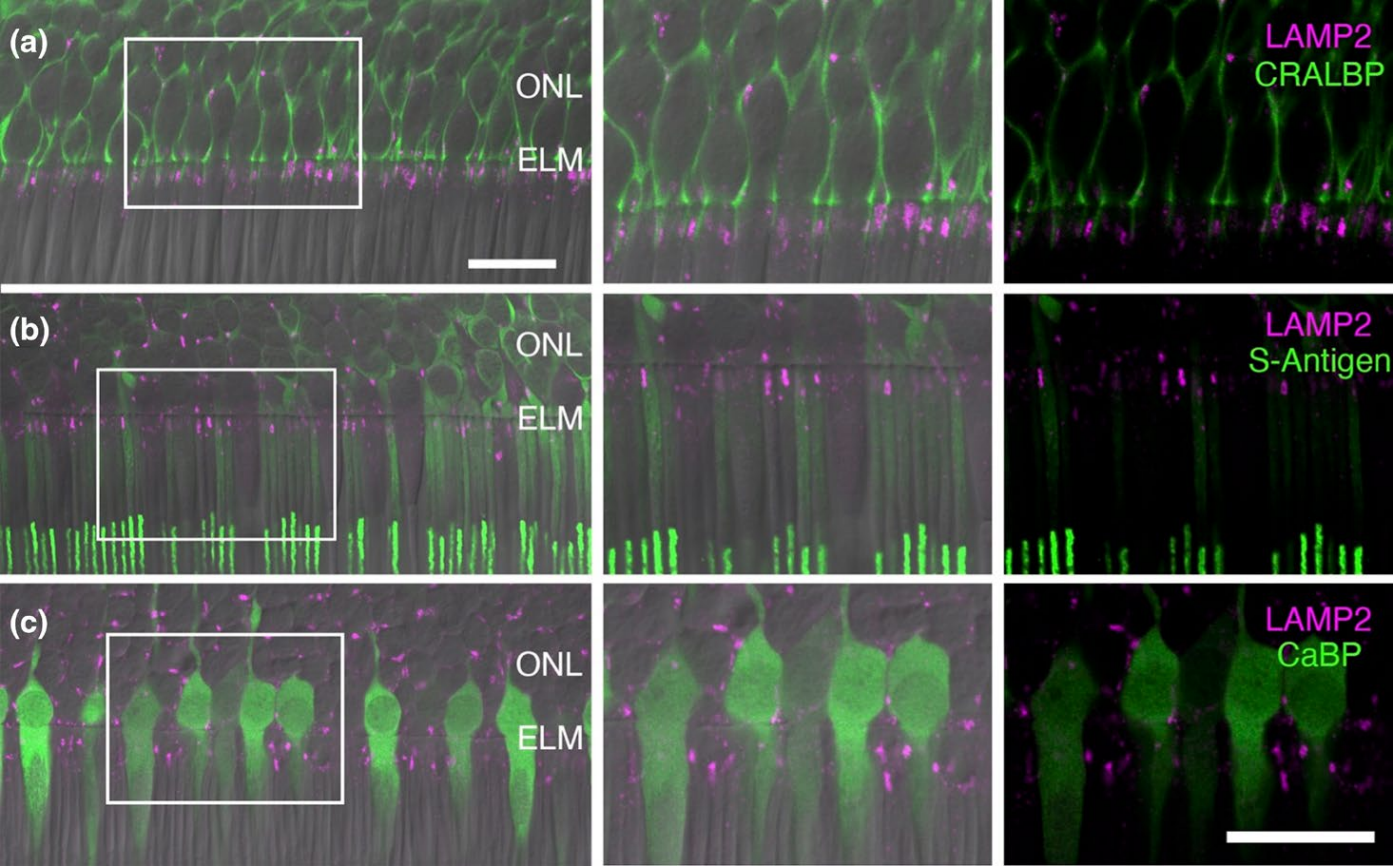
LAMP2 expression in a postmortem normal human retina. Confocal images of vertical vibratome sections through the macula. The sections were processed with antibodies against LAMP2 (magenta) and CRALBP to label Müller cells (a), S‐antigen (green) to label rods (b) or calbindin (CaBP, green) to label cones (c). Boxes in the left panels indicate the regions shown in the middle and right panels at higher resolution. The middle panels show immunofluorescence staining together with differential interference contrast optics to reveal retinal layers. Punctate LAMP2 staining was located within the outer nuclear layer (ONL) near the apical region of the Müller cells and in the inner segments of the rod but not cone photoreceptors external to the external limiting membrane (ELM). Scale bar shown in A = 20 μm, applies to the left panels; scale bar shown in C = 20 μm, applies to middle and right panels.

**FIGURE 7:**
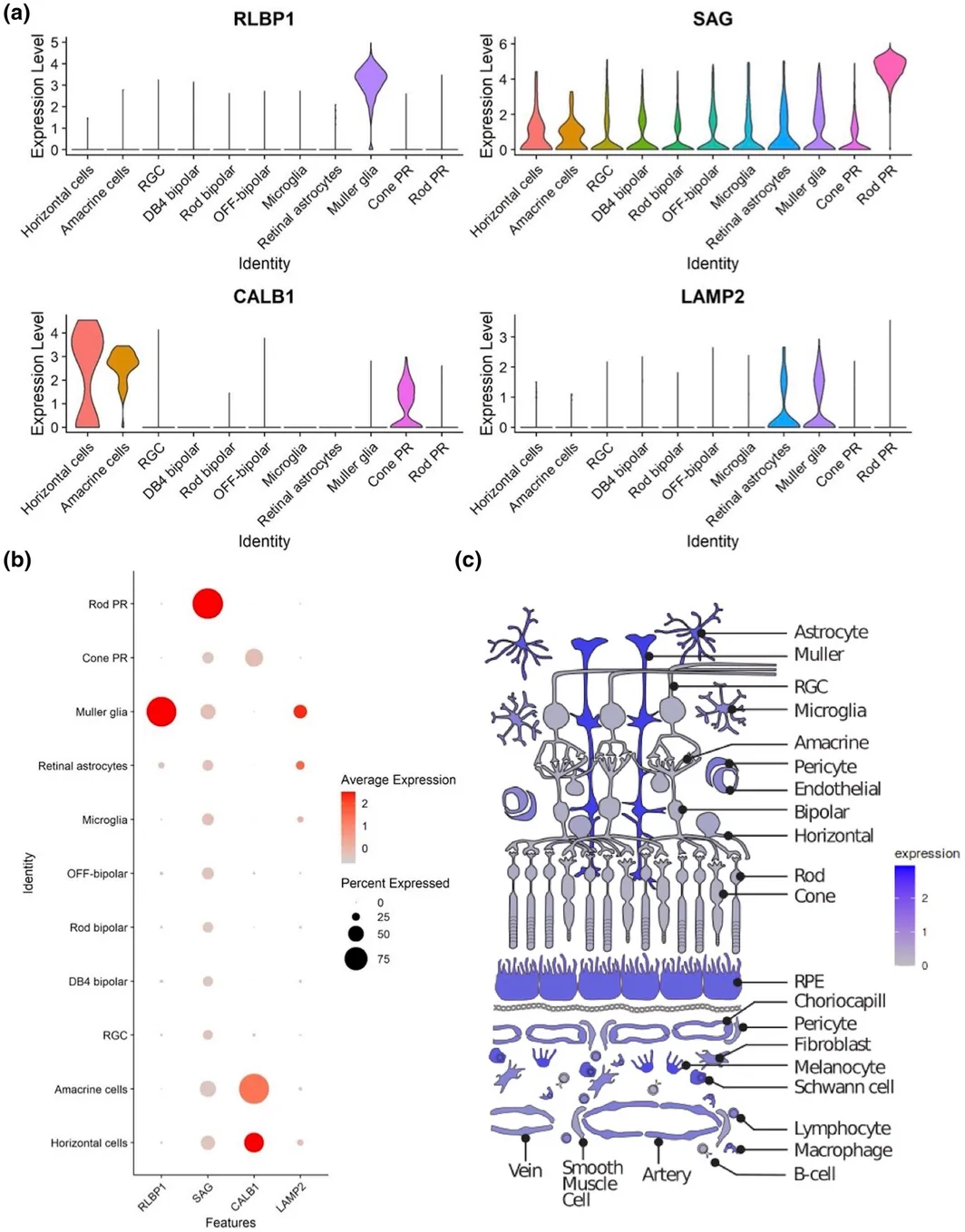
Single cell gene expression profile of Müller cell (*RLBP1*), rod (*SAG*) and cone (*CALB1*) photoreceptor markers compared to *LAMP2* across various retinal cell types shows predominance of LAMP2 expression in retinal astrocytes and Müller glia. Violin (a) and dot (b) plots showed the normalised expression levels of the 4 retinal markers used in histology. Violin plots showed distribution of single cell expression levels across various retinal cell types. Data were extracted from the single‐cell transcriptome study (Lukowski et al., 2019). LAMP2 expression in the retina, RPE and choroid shown in a heat scale extracted from the single cell transcriptome data (Voigt et al., 2020) available through https://singlecell‐eye.org (c).

## 4 DISCUSSION

Hyperreflective deposits within the ONL in *LAMP2* have been previously described (Dao et al., 2024; Emfietzoglou et al., 2026; Kousal et al., 2021). A recent paper found ONL hyperreflectivity was highly prevalent in 39 of 40 available OCT scans from 29 genetically confirmed DD cases (Emfietzoglou et al., 2026). However, our study described the radial distribution of these deposits with en face OCT known as the RORR sign. This finding was reproducible across 18 eyes in 9 cases. The en face maps confirmed these hyperreflective lesions were located throughout the central macula without foveal sparing. On OCT these deposits extended from the outer part of the ONL and stopped at the ELM with no extension observed into the myoid. This was intriguing given the radial nature of the peripheral RPE depigmentation observed with multimodal imaging. We hypothesise the radial nature to be attributed to X‐chromosome inactivation within the Müller glia or photoreceptor cells.

It has been well documented that individuals harbouring pathogenic *LAMP2* variants develop radial streaks of RPE depigmentation (Dao et al., 2024; Hasegawa et al., 2022; Kousal et al., 2021; Narayan et al., 2024; Nishino et al., 2000; O'Neil et al., 2022; Schorderet et al., 2007; Thompson et al., 2016). Our study compared the peripheral retinal pigmentary changes in LAMP2 to other X‐linked carrier retinopathies. Carriers of X‐linked ocular albinism (pathogenic *GPR143* variants) show peripheral streaks of hypopigmentation known as ‘mud‐splatter’ (Khan et al., 2018). A continuous streak of hypopigmented cells should not occur by chance alone (Khan et al., 2018). Instead, the authors suggested these streaks arise from a common RPE progenitor cell that has inactivated the wildtype X‐chromosome. Thus, we propose the radial pattern observed in both *LAMP2* and *GPR143* carriers may both be explained by the migration of developing RPE cells derived from a common progenitor. Although these radial streaks have been documented previously, our study examines peripheral OCT slices through these streaks showing transmission artefact due to RPE depigmentation. We observed intact outer retinal layers in regions of RPE depigmentation.

*LAMP2* produces 3 isoforms namely, LAMP2a, LAMP2b and LAMP2c. A previous study has shown both LAMP2a and LAMP2b were present in a human RPE cell line (Schorderet et al., 2007). Although that study confirmed strong expression of LAMP2 in the RPE, the other retinal layers were below detection levels. Whilst our histologic work was performed on one normal human donor eye and supports LAMP2 retinal expression, it does not confirm the pathological origins of the RORR sign or previous OCT findings (Emfietzoglou et al., 2026; Kousal et al., 2021). Further support for LAMP2 expression within the neuroretina comes from a single‐cell transcriptome study (Lukowski et al., 2019). Analysis of the data provided in their supplementary material showed LAMP2 had the highest expression profile in the Müller glia when compared to other retinal cell types including rods, cones, bipolar cells, retinal ganglion, amacrine and microglia cells. This distribution was also supported by the single cell RNA sequencing data (Voigt et al., 2020) available through the interactive Spectacle website (updated in 2022). Notably, there was some expression in the rod photoreceptors which was consistent with our finding of LAMP2 within inner segment myoids. Another study investigated LAMP2 expression in mice and found significantly higher levels in the RPE compared to the retina (Notomi et al., 2019).

As *LAMP2* remains a very rare disease this study was limited by its small sample size and only one male case. Thus, any generalisations should be interpreted with caution particularly given the marked phenotypic variability seen in females due to tissue patterns of X‐chromosome inactivation. Our data suggest macula‐to‐periphery variation in lysosomal flux across retinal cell populations. Future histological studies, including electron microscopy, will be required to determine what these hyperreflective lesions are comprised of. A larger sample size will help more reliably establish the specificity and sensitivity of the RORR sign. It is notable however that our youngest case showed the RORR sign at only 12 years of age. It should be noted that the cases in the present study were mostly female, and the only male was presumed mosaic. Thus, it is not clear if our findings would be generalisable to truly hemizygous males. Furthermore, given each patient may have had different foveal anatomy we cannot guarantee the RORR sign was observed in the same retinal layers.

This study expands the phenotypic spectrum of *LAMP2* by demonstrating the RORR sign using en face OCT. The RORR sign could serve as a valuable diagnostic marker which can guide molecular investigations of *LAMP2* variants. We report histological evidence of *LAMP2* staining in the neuroretina, specifically the apical domain of the Müller glia.

## Supporting information


Data S1.

## Data Availability

The data supporting the results of this manuscript are available upon request to the corresponding author (FKC).
