# Supplementary material for: Radial outer retina reflectivity (RORR) sign in LAMP2 ‐associated retinopathy
Source: Acta Ophthalmol. 2026 Jun 28;104(8):e960–72. doi: 10.1111/aos.70178 (PMC13751869; doi:10.1111/aos.70178)
Supplement: Supplementary file 1 [file AOS-104-e960-s001.pdf]

Grader 1

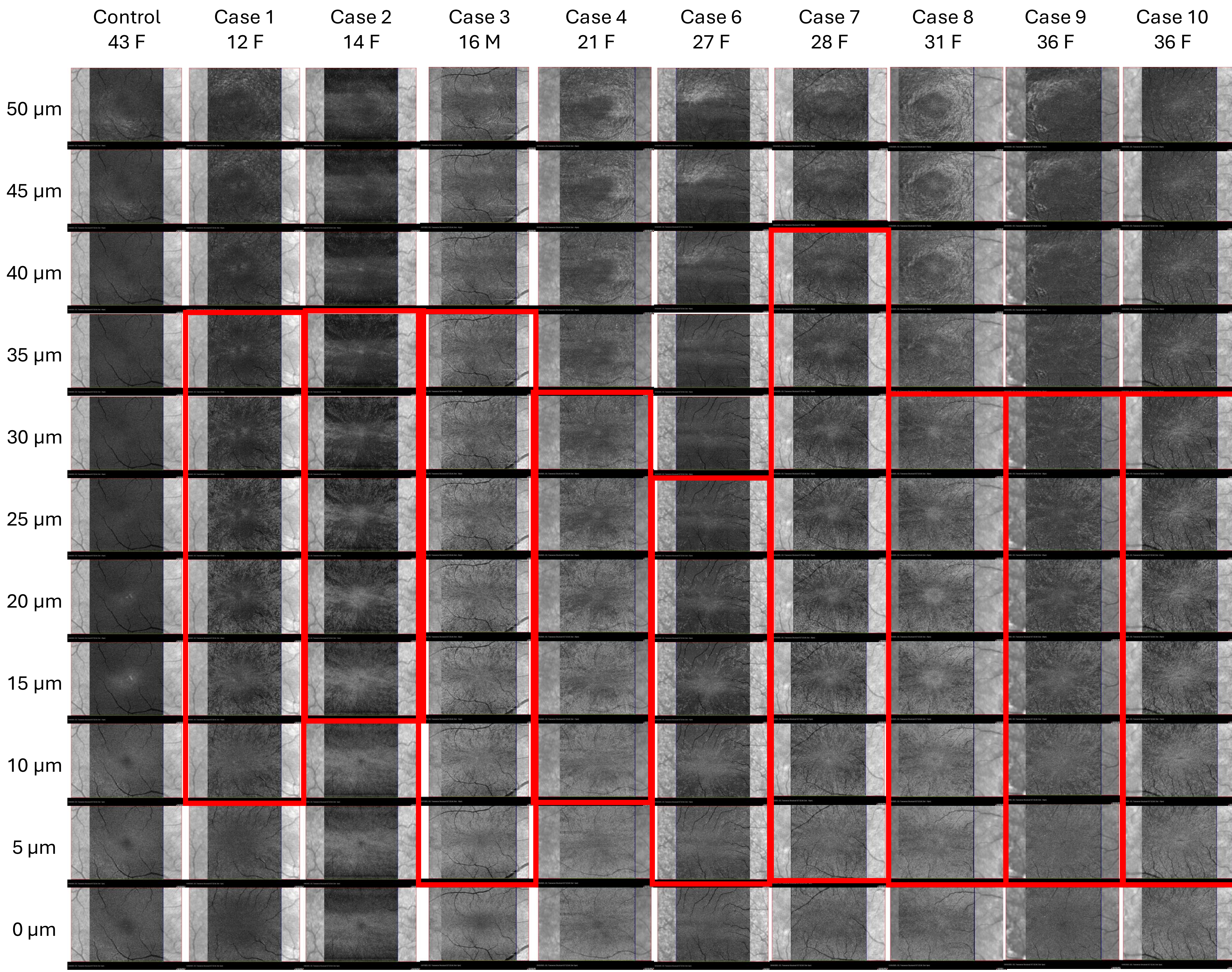
Control 43 F
Case 1 12 F
Case 2 14 F
Case 3 16 M
Case 4 21 F
Case 6 27 F
Case 7 28 F
Case 8 31 F
Case 9 36 F
Case 10 36 F
50 µm
45 µm
40 µm
35 µm
30 µm
25 µm
20 µm
15 µm
10 µm
5 µm
0 µm

Grader 2

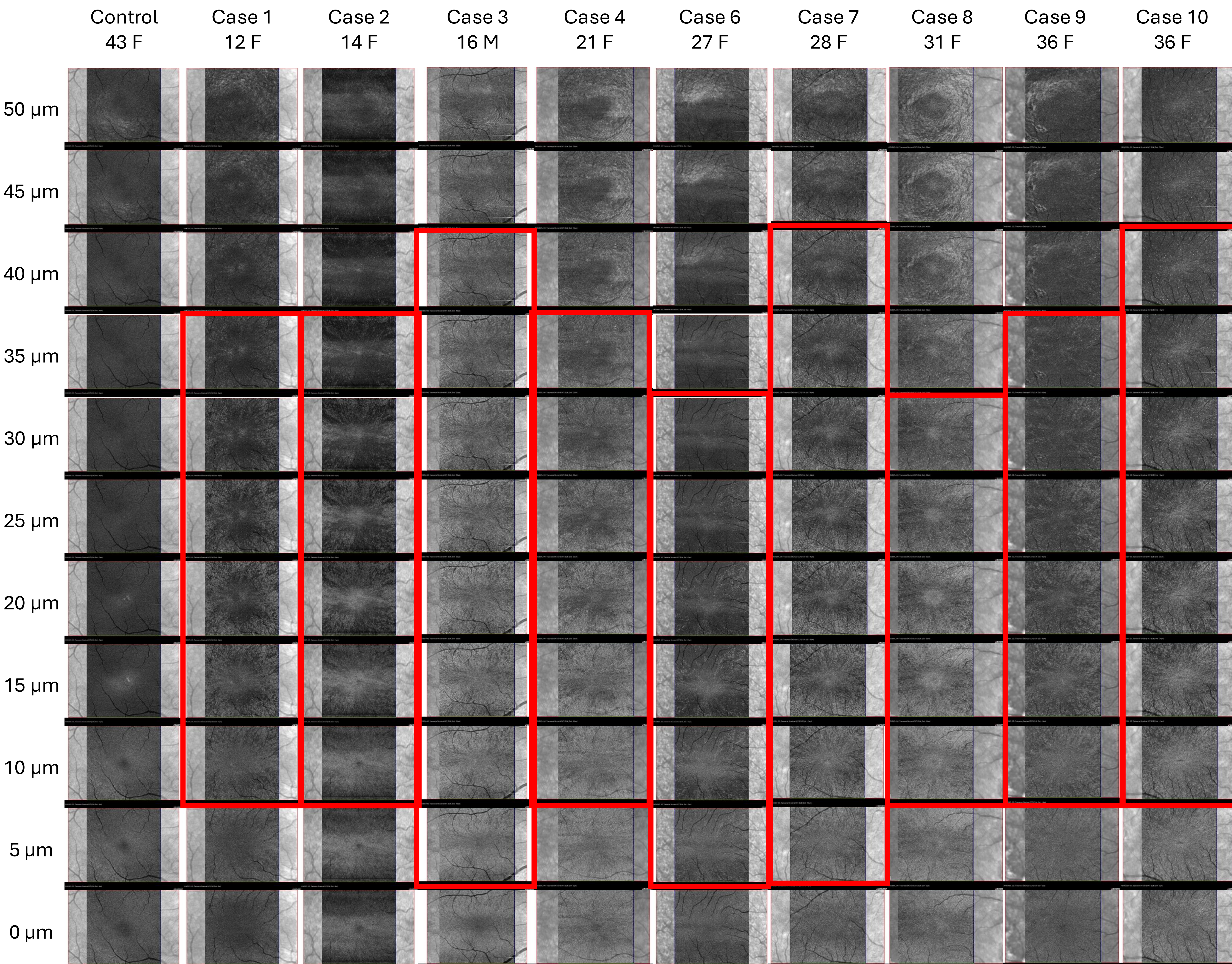
Control
43 F
Case 1
12 F
Case 2
14 F
Case 3
16 M
Case 4
21 F
Case 6
27 F
Case 7
28 F
Case 8
31 F
Case 9
36 F
Case 10
36 F
50 µm
45 µm
40 µm
35 µm
30 µm
25 µm
20 µm
15 µm
10 µm
5 µm
0 µm

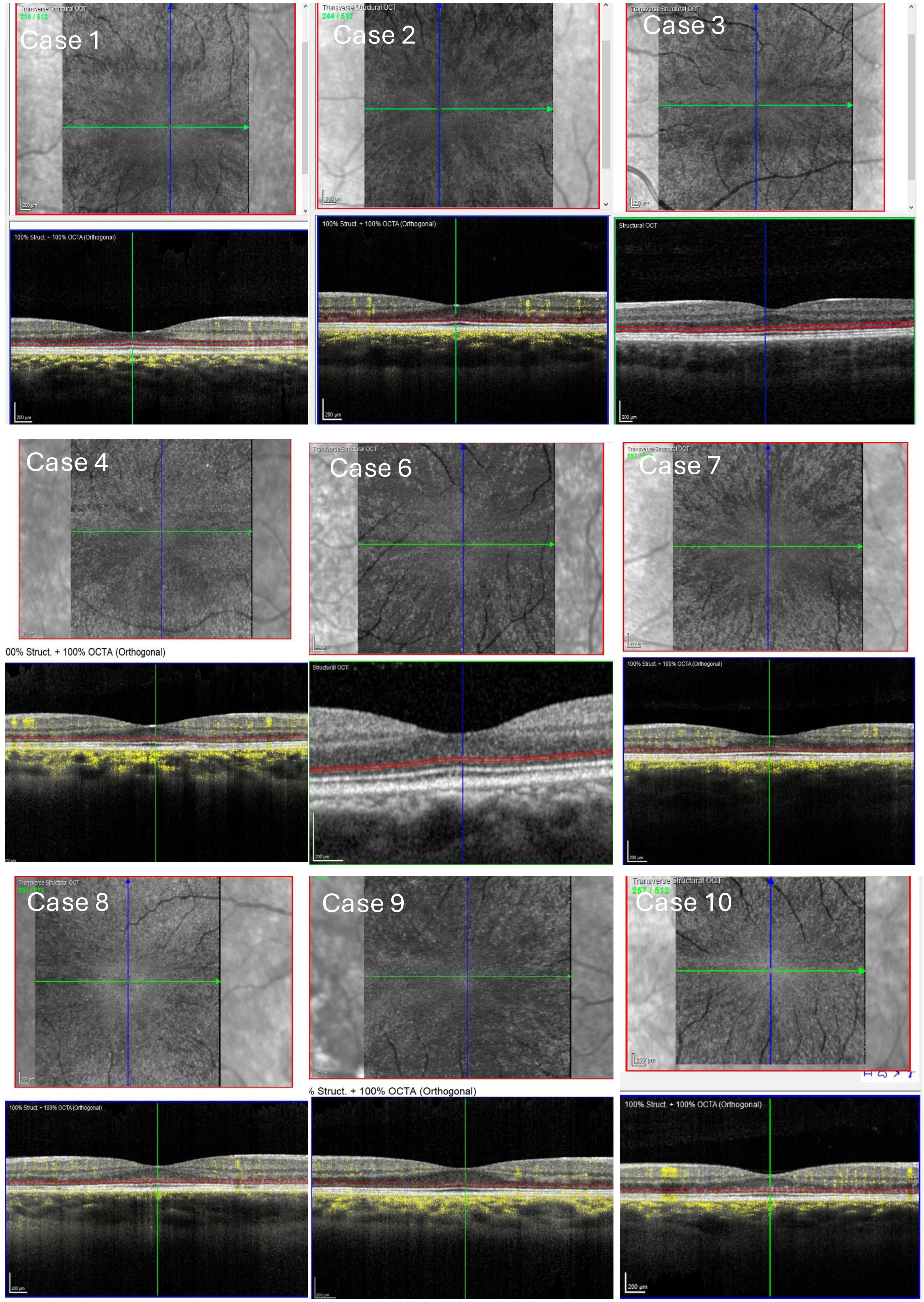
Case 1
Case 2
Case 3
Case 4
Case 6
Case 7
00% Struct. + 100% OCTA (Orthogonal)
Case 8
Case 9
Case 10
% Struct. + 100% OCTA (Orthogonal)
100% Struct. + 100% OCTA (Orthogonal)
